# Denosumab alleviates intervertebral disc degeneration adjacent to lumbar fusion by inhibiting endplate osteochondral remodeling and vertebral osteoporosis in ovariectomized rats

**DOI:** 10.1186/s13075-021-02525-8

**Published:** 2021-05-28

**Authors:** Qi Sun, Fa-Ming Tian, Fang Liu, Jia-Kang Fang, Yun-Peng Hu, Qiang-Qiang Lian, Zhuang Zhou, Liu Zhang

**Affiliations:** 1 Department of Orthopedic Surgery, Hebei Medical University, Shijiazhuang, People’s Republic of China; 2 Medical Research Center, North China University of Science and Technology, Tangshan, People’s Republic of China; 3 Department of Bone and Soft Tissue Oncology, The Third Hospital of Hebei Medical University, Shijiazhuang, People’s Republic of China; 4 Department of Orthopedic Surgery, Hebei Medical University, 361 Zhongshan ERoad, Hebei, 050000 Shijiazhuang People’s Republic of China

**Keywords:** Osteoporosis, Ovariectomy, Vertebral, Endplate, Osteochondral remodeling

## Abstract

**Background:**

Although adjacent segmental intervertebral disc degeneration (ASDD) is one of the most common complications after lumbar fusion, its exact mechanism remains unclear. As an antibody to RANKL, denosumab (Dmab) effectively reduces bone resorption and stimulates bone formation, which can increase bone mineral density (BMD) and improve osteoporosis. However, it has not been confirmed whether Dmab has a reversing or retarding effect on ASDD.

**Methods:**

Three-month-old female Sprague-Dawley rats that underwent L4–L5 posterolateral lumbar fusion (PLF) with spinous-process wire fixation 4 weeks after bilateral ovariectomy (OVX) surgery were given Dmab 4 weeks after PLF surgery (OVX+PLF+Dmab group). In addition, the following control groups were defined: Sham, OVX, PLF, and OVX+PLF (*n*=12 each). Next, manual palpation and X-ray were used to evaluate the state of lumbar fusion. The bone microstructure in the lumbar vertebra and endplate as well as the disc height index (DHI) of L5/6 was evaluated by microcomputed tomography (μCT). The characteristic alterations of ASDD were identified via Safranin-O green staining. Osteoclasts were detected using tartrate-resistant acid phosphatase (TRAP) staining, and the biomechanical properties of vertebrae were evaluated. Aggrecan (Agg), metalloproteinase-13 (MMP-13), and a disintegrin and metalloproteinase with thrombospondin motifs 4 (ADAMTS-4) expression in the intervertebral disc were detected by immunohistochemistry and real-time polymerase chain reaction (RT-PCR) analysis. In addition, the expression of CD24 and Sox-9 was assessed by immunohistochemistry.

**Results:**

Manual palpation showed clear evidence of the fused segment’s immobility. Compared to the OVX+PLF group, more new bone formation was observed by X-ray examination in the OVX+PLF+Dmab group. Dmab significantly alleviated ASDD by retaining disc height index (DHI), decreasing endplate porosity, and increasing vertebral biomechanical properties and BMD. TRAP staining results showed a significantly decreased number of active osteoclasts after Dmab treatment, especially in subchondral bone and cartilaginous endplates. Moreover, the protein and mRNA expression results in discs (IVDs) showed that Dmab not only inhibited matrix degradation by decreasing MMP-13 and ADAMTS-4 but also promoted matrix synthesis by increasing Agg. Dmab maintained the number of notochord cells by increasing CD24 but reducing Sox-9.

**Conclusions:**

These results suggest that Dmab may be a novel therapeutic target for ASDD treatment.

## Introduction

Lumbar fusion is a widely used, principal operation for spinal diseases that effectively relieves symptoms of lower back pain [1]. Adjacent segmental intervertebral disc degeneration (ASDD), an important and prevalent complication of lumbar fusion, seriously affects long-term clinical outcomes for patients [2], requires secondary operations, and increases health care costs [1]. Clinical treatment methods for ASDD are currently limited because they cannot fully relieve the symptoms induced by degeneration of the intervertebral disc, and it cannot fundamentally reverse ASDD. Therefore, it is necessary to develop new effective therapeutic strategies to ameliorate ASDD progression.

Although the etiology of ASDD is complex and insufficiently understood, there is accumulating evidence that endplate osteochondral remodeling and vertebral osteoporosis might play critical roles in ASDD [3]. The intervertebral disc (IVD) is composed of the central nucleus pulposus (NP), surrounded by the collagenous annulus fibrosus (AF) and two endplates (EP) located above and below the NP and AF [4]. Commonly, the gelatinous NP is the main functional component of IVD, and the cellular changes and degradation of the NP tissue’s extracellular matrix are major causes of disc degeneration [5]. Anatomically, the vertebrae and IVD are combined into a bundle to form the spine’s motor segment. From a mechanical and biological point of view, they are closely connected and considered to be a functional unit. Therefore, the health of the vertebral bone and health of IVDs are closely related [6]. In addition, IVDs are the largest source of avascular tissue in the human body; therefore, a bone marrow channel between the EP, NP, and AF is essential for nutrient absorption and metabolite exchange in the disc. The cells in the outer annulus of the AF receive nutrition primarily from the circumferential pathway while the NP cells almost completely rely on the nutrition provided by the vertebral capillary bed adjacent to the EP [4, 7–9]. Under cyclic loading, the nutrient supply of the IVD and discharge of metabolic waste depend on diffusion and fluid flow, which is mainly affected by endplate penetration [10].

Osteoporosis, which is prevalent in postmenopausal and older populations, is characterized by decreased bone mass and reduced bone mineral density (BMD), accompanied by the destruction of bone microstructure, resulting in increased bone fragility and fracture risk [11]. Recent studies have confirmed that osteoporosis is not only an important risk factor for complications of vertebral nonunion after spinal fusion but also the main inductor of ASDD [6].

Different from other anti-osteoporosis drugs, denosumab (Dmab) is a human monoclonal immunoglobulin G2 (IgG2) antibody that competitively binds to receptor activator of nuclear factor kappa-B ligand (RANKL), preventing the binding of RANKL to its osteoclast-derived receptor (RANK), thereby inhibiting osteoclast-induced bone resorption activity [12]. Dempster et al. [13] demonstrated that Dmab preserved femoral neck cartilage remodeling while retaining modeling-based bone formation, and after Dmab treatment, BMD increased continuously over time. Recent studies have shown that EP cartilage remodeling and vertebral osteoporosis may be important pathogenetic factors of ASDD.

However, it is still unknown whether Dmab can inhibit the remodeling of adjacent intervertebral disc endplate cartilage in lumbar fusion and whether it can improve the prognosis of osteoporotic lumbar fusion in elderly postmenopausal women. In the present study, an ovariectomized rat model was used to investigate the effect of subcutaneous Dmab administration on ASDD after lumbar fusion, providing a basis for the clinical treatment of ASDD.

## Materials and methods

### Experimental animals

All experimental protocols were approved by the Institutional Animal Care and Use Committee. A total of 60 three-month-old female Sprague-Dawley rats (each weighing 259 ± 18 g) (Vital River Experimental Animal Technical Co., Ltd., Beijing, China) were used for this study. The animals were kept in a ventilated environment with a 12:12-h light–dark cycle at a constant temperature of 21°C.

### Operation procedures, groups, and study design

After anesthesia, each rat underwent either sham operation (Sham, only a skin incision was made and then sutured) or bilateral ovariectomy (OVX). Four weeks after OVX surgery, an experimental model of posterolateral spinal fusion (PLF) was established via an intertransverse process fusion using an autologous iliac graft with wire fixation at the L4–L5 spinous processes. Finally, the fascia and skin were closed. The surgical procedure replicated the previously validated model described by Boden and colleagues [14]. All animals were given prophylactic antibiotics (penicillin-G; 40,000 U) beginning soon after surgery and lasting for 3 days. Then, rats received subcutaneous administration of either vehicle or 0.25 mg/kg/day Dmab 5 days per week for 4 weeks in the following groups (*n*=12 each): (1) Sham, (2) OVX, (3) PLF, (4) OVX+PLF, and (5) OVX+PLF+Dmab. Weights were recorded weekly, and Dmab doses were adjusted accordingly.

Four weeks after PLF, radiographic evaluation and manual palpation were performed to evaluate the lumbar fusion. Next, the L3–L6 segment of the spine was removed, the attached muscles were removed, and the fusion was evaluated manually by palpation. Half of the samples (*n*=6, each group) were used for histological and immunohistochemical (L5–L6) and real-time polymerase chain reaction (RT-PCR) analysis (L3–L4). The other half (*n*=6, each group) underwent microcomputed tomography (μCT) scanning and testing of the mechanical properties of the vertebral body.

### Evaluation of posterolateral lumbar fusion

Four weeks after PLF, the animals were anesthetized and the fusion was evaluated with a soft radiograph (DR7500 System, Kodak, USA) in the anteroposterior plane and analyzed by an experienced radiologist blinded to the study, which was performed in accordance with the criteria established by O’Loughlin et al. [14]. The animals were euthanized 8 weeks after the X-ray examination, after which the manual palpation of the fusion site (L4–L5 segment) was performed to assess the fusion status as described by Abe et al. [15]. This process is considered to be the gold standard for detecting pseudoarticulation formation or stabilization of the fixation [16]. Each fusion site was evaluated by three independent observers in a blinded fashion.

### μCT analysis

The L5–L6 segment was scanned using a SkyScan 1176 microcomputed tomography system in accordance with a previously described protocol [6]. In brief, the scanner was operated at a voltage of 80 kV, a current of 313 μA, and a resolution of 18 μm. Images were reconstructed using NRecon v1.6 software. Three-dimensional (3D) reconstruction images were obtained using CTvox v3.0. The regions of interest (ROI) of vertebrae and endplates as well as the disc height index (DHI) were chosen using CTAn v1.14 and DataViewer v.1.5.

After excluding the cortical bone, the transverse images of L6 vertebrae were used to measure the vertebral bodies. The IVD and vertebral bone heights were measured between L5 and L6 at the mid-sagittal plane, and the DHI was calculated using the following equation: DHI = (disc height from anterior+ disc height from posterior)/(vertebral bone height from anterior + vertebral bone height from posterior) [17]. The ROI of the cartilage endplate was restricted to the visible bone plate that covered the vertebrae. The 3D structural parameters of the vertebrae included bone volume fraction (BV/TV), trabecular thickness (Tb.Th), trabecular number (Tb.N), and trabecular separation (Tb.Sp). The parameters of the endplate included BMD, number of closed pores (Po.N (cl); representing the number of pores with a closed cavity in the endplate structure), open porosity (Po (op); open pore volume over total pore volume), and total volume of pore space (Po.V (tot)).

### Vertebral body compression experiment

Prior to the beginning of the experiment, all vertebral appendages, including transverse processes, superior and inferior articular processes, pedicles, and lamina, were removed, and only the vertebral body was retained. The upper and lower parts of the vertebral body were polished and standardized to a height of about 5 mm and were perpendicular to the longitudinal axis of the vertebral body. It was assumed that the vertebral body was cylindrical, and the average diameter and body boundary area of the standard components were calculated based on the anterior and posterior diameter of each vertebral body. The lower end of the vertebral body was fixed on the test platform with glue, and the vertebral body was axially compressed at a speed of 4 mm/min until fracture. The maximum compression load and energy absorption values were recorded, and the maximum stress was calculated as the ratio of maximum pressure and body interface area (maximum compressive strength of the vertebral body). During the experiment, normal saline was used to keep the vertebral specimens in a moist state.

### Histology and immunohistochemistry examinations

After fixation in 10% neutral paraformaldehyde for 48 h, the L5–L6 segments of the lumbar spine (including the IVD) were decalcified in 10% EDTA-2Na for 3 months at room temperature. The decalcified samples were dehydrated and embedded in paraffin. They were subsequently cut into 8-μm-thick sections to perform safranin O and fast green staining, tartrate-resistant acid phosphatase (TRAP) staining, and immunohistochemistry. The images were captured by a microscope system (BX53; Olympus, Tokyo, Japan). The degenerative changes in the L5–L6 segment were assessed using the disc degeneration assessment scoring system described by Masuda et al. [18]. AF and NP were scored respectively, ultimately combined to obtain the IVD score.

Safranin-O green staining was performed in accordance with the instructions of the reagent kits (Servicebio Biological Technology Co., Ltd., China). TRAP staining was handled in accordance with the staining kit protocol (Solarbio Science & Technology Co. Ltd., China).

To observe the tissue expression of aggrecan (Agg), metalloproteinase-13 (MMP-13), and a disintegrin and metalloproteinase with thrombospondin motifs 4 (ADAMTS-4), 8-μm-thick sections were deparaffinized, rehydrated, and immunostained. In brief, after antigen retrieval and inactivation of endogenous peroxidase, the sections were incubated overnight at 4°C with primary antibodies: Agg (1:500 dilution; Cat. No. GTX54920; GeneTex Inc. USA), MMP-13 (1:200 dilution; Cat. No. GTX55707; GeneTex Inc., USA), ADAMTS-4 (1:200 dilution; Cat. No. ab185722; Abcam Inc., USA), CD24 (1:100 dilution; Cat. No. bs-0528R; Bioss Inc., Beijing, China), and SOX-9(1:100 dilution; Cat. No. ET1611-56; HuaAn., HangZhou, China). The next day, the sections were incubated with a biotinylated secondary antibody and a streptavidin-biotin complex peroxidase solution. Diaminobenzidine (DAB) chromogen was applied, and the sections were counterstained with hematoxylin.

All images were captured by a BX53 microscope system (Olympus, Tokyo, Japan). The integrated optical density (IOD) values of each factor were semiquantitatively analyzed using Imaging Pro Plus 6.0 software (Media Cybernetics, Rockville, MD, USA). The intensity of positive staining in the ROI was calculated and defined as the sum of integrated optical density (IOD), and the area the of ROI was also calculated. The average IOD of specific proteins, reported as IOD/mm^2^, was defined as the sum of IOD divided by area of ROI. The final result used for the statistical analysis was the average of values calculated by two individuals who scored the sections in a blinded manner.

### RT-PCR

NP samples from the L3–L4 IVD were obtained for this analysis. A Gene Amp 7,700 Sequence Detection System (Applied Biosystems, Foster City, CA,USA) and SYBER® Premix Ex Taq™II kit (Takara, Kusatsu, Japan) were used to perform RT-PCR. The primers for the selected genes are listed in Table 1. GAPDH was used as an endogenous control. The changes of relative mRNA transcript levels were reported using the 2 (-Delta C(T)) method as previously described [19]. The experiment was repeated at least three times to ensure accuracy.

**Table 1:** Sequences of primers used for RT-PCR

Gene	Forward primer (5′-3′)	Reverse primers (5′-3′)
GAPDH	GGGGAGCCAAAAGGGTCATCATCT	GAGGGGCCATCCACAGTCTTCT
Aggrecan	GAAGTGGCGTCCAAACCAAC	AGCTGGTAATTGCAGGGGAC
ADAMTS-4	CGTTCCGCTCCTGTAACACT	TTGAAGAGGTCGGTTCGGTG
MMP-13	TGCTGCATACGAGCATCCAT	TTCCCCGTGTCCTCAAAGTG

### Statistical analysis

All data were analyzed using SPSS software (SPSS, Chicago, IL, USA), and the results were expressed as the mean ± standard deviation (SD). The Shapiro-Wilk test for normality and Bartlett’s test for homogeneity of variance were performed. One-way analysis of variance (ANOVA) and Fisher’s protected least significant difference test were used to determine the statistically significant differences between the groups. The results of the radiography scores were analyzed using the Kruskal-Wallis test. *P* < 0.05 was considered to indicate statistical significance.

## Results

### Evaluation of lumbar fusion

**Fig. 1:**
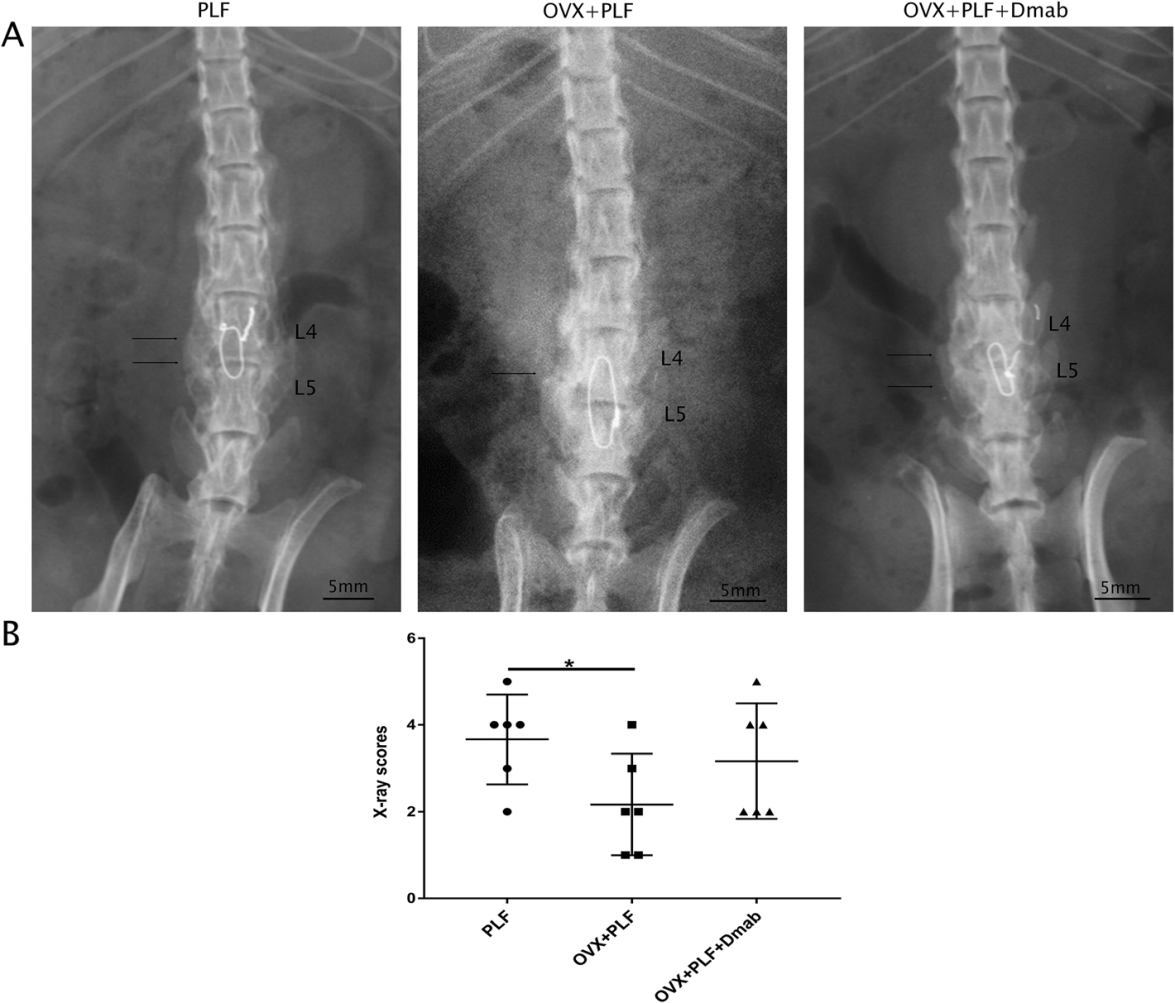
Radiographic evaluation of lumbar fusion at 4 weeks post-PLF. **a** Representative radiographic images from the three groups. PLF of the L4–L5 segments was performed via intertransverse process fusion with an autologous iliac bone graft and spinous-process wire fixation. Compared with the OVX+PLF group, the OVX+PLF+Dmab group showed higher radiographic density with more new bone formation at the fusion site (thin arrow indicates new bone formation). **b** X-ray scores of lumbar fusion. Note: ^*^P<0.05; scale bars = 5 mm as indicated

To evaluate the fusion of the L4-L5 segment, the segment was examined by X-ray and manual palpation. As shown in Fig. 1, the three groups all showed adequate wire fixation. Compared to OVX+PLF group, the fusion site of the OVX+PLF+Dmab group showed higher radiographic density and increased formation of new osteotyluses, but there was no obvious changes between the OVX+PLF and OVX+PLF+Dmab groups. The fusion score of the OVX+PLF group was significantly lower than that of the PLF groups. OVX+PLF+Dmab group had a higher score than the OVX+PLF group, but without reaching statistical significance.

During manual palpation, the wire on the L4-L5 spinous processes of all rats who underwent PLF surgery was found to be well connected to the spinous processes, and there was no detectable movement at the fusion level. These results revealed adequate lumbar fusion of rats after PLF.

### μCT parameters of the L5-L6 segments

**Fig. 2:**
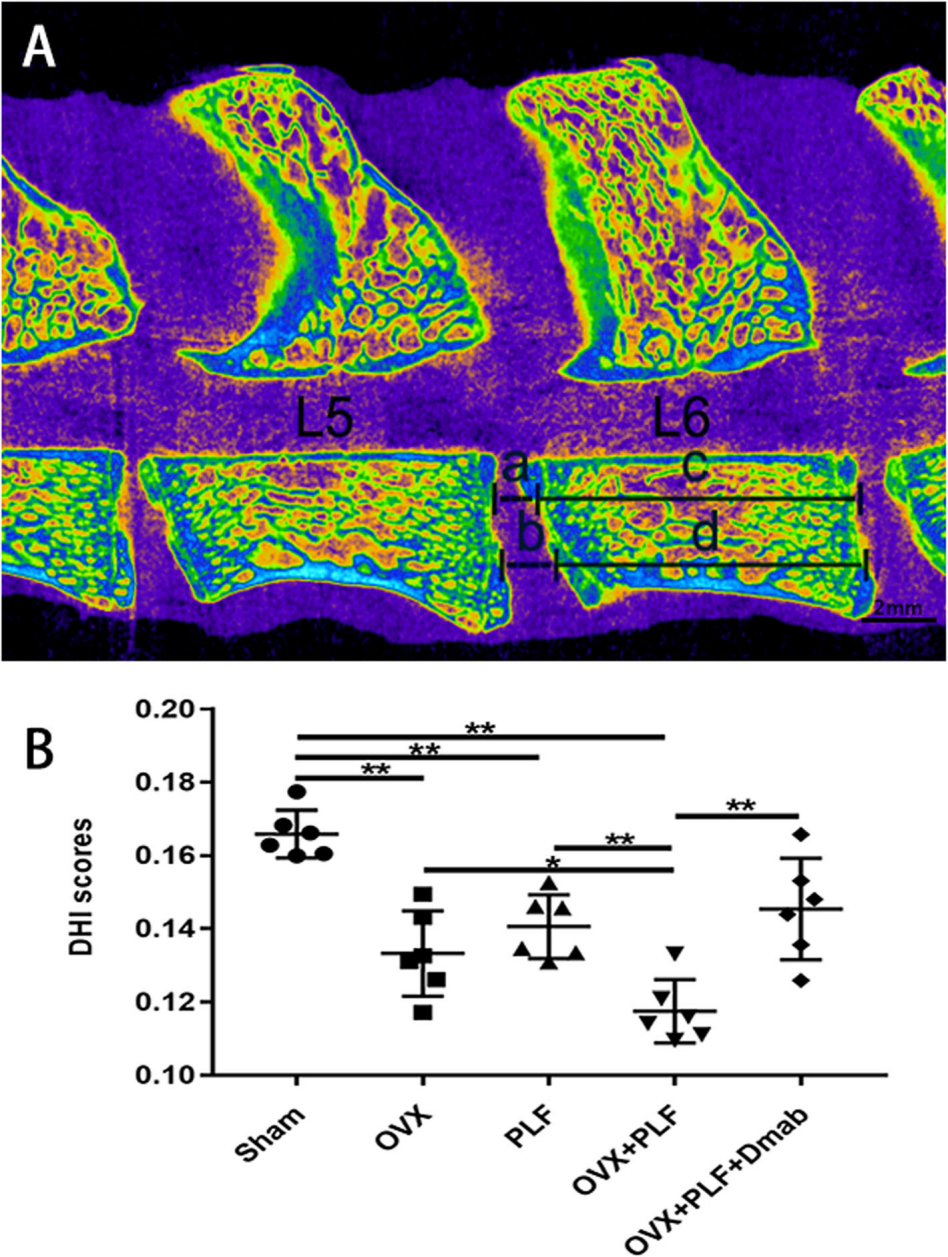
Representative micro-CT image of the lumbar spine used to quantify the DHI between the L5 and L6 vertebrae, calculated based on measurements of the adjacent L6 vertebra (**a**). DHI was calculated using the following equation: DHI= (a+b)/(c+d). **b** The results of DHI scores. Note: ^*^P < 0.05, ^**^P < 0.01; scale bars = 2 mm as indicated

To quantify vertebral bone and endplate microarchitecture, μCT analysis was performed on L5–L6 segments. Since loss of IVD height is used as an alternative predictor of intervertebral disc degeneration, the DHI of L5–L6 were measured (Fig. 2a). DHI was significantly lower in the OVX and OVX+PLF groups than in the Sham group, while there was no significant difference between the OVX and PLF groups. DHI was significantly higher in the OVX+PLF+Dmab group compared with the OVX+PLF group (Fig. 2b).

**Fig. 3:**
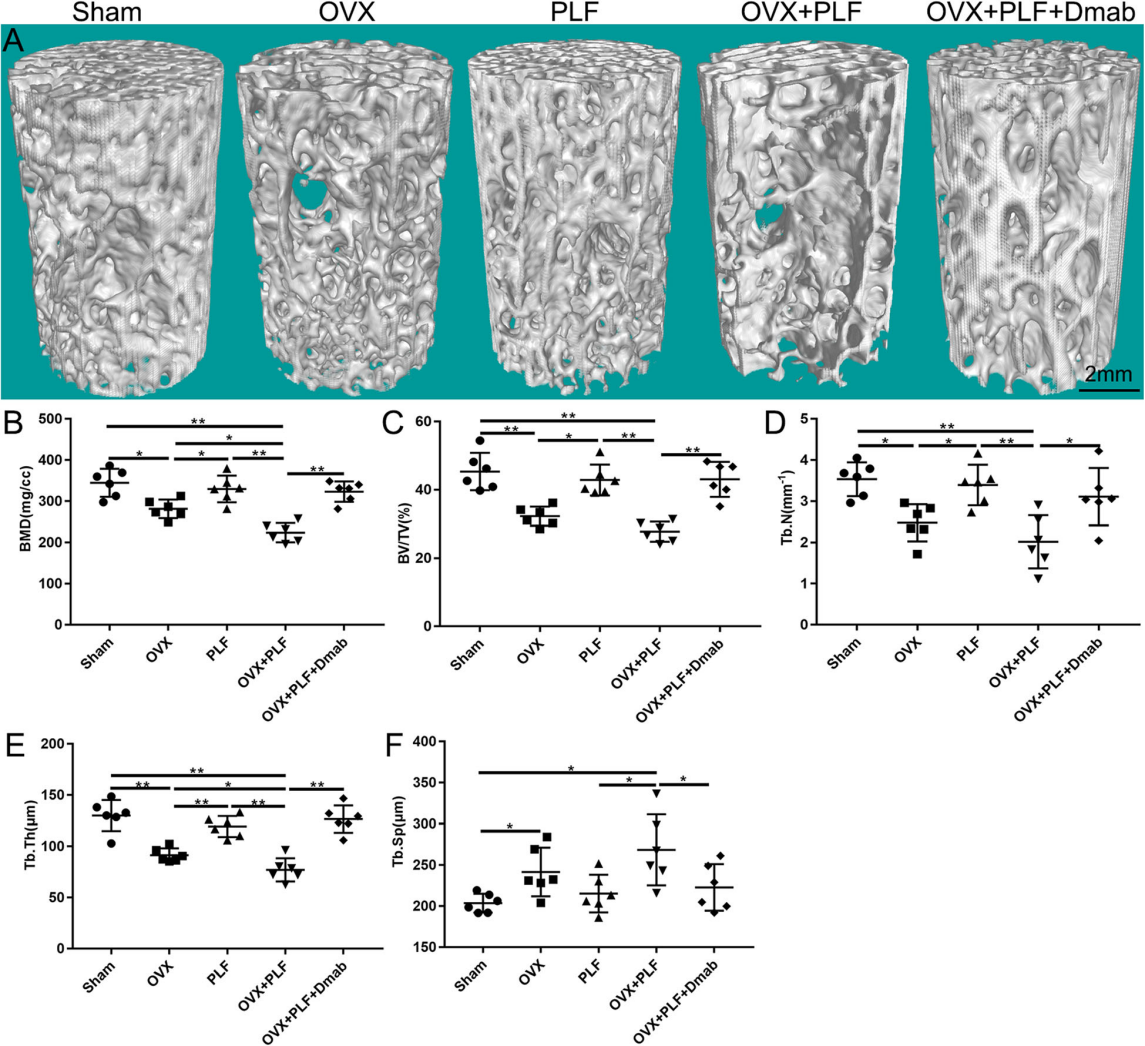
Representative micro-CT image of L6 vertebra (**a**). Compared to the Sham group, the trabeculae were sparser and the width of the canal between trabeculae was higher in the OVX and OVX+PLF groups; a large area of bone trabeculae was missing in the OVX+PLF group. The parameters of L6 trabecular bone (**b**–**f**). The results of L6 vertebral BMD, bone volume (BV)/total volume (TV), trabecular number (Tb.N; mm-1), trabecular thickness (Tb.Th; μm), and trabecular separation (Tb.Sp; μm) in different groups. Note: ^*^P < 0.05, ^**^P < 0.01; scale bars = 2 mm as indicated

As shown in Fig. 3a, compared to the Sham group, trabeculae were sparser, and the width of the canal between trabeculae was higher in the OVX and OVX+PLF groups. Additionally, a large area of bone trabeculae was missing in the OVX+PLF group. The OVX+PLF+Dmab group showed higher trabecular thickness, lower tube diameter between trabeculae, and more compact trabecular structure compared to the OVX+PLF group. μCT evaluation of L6 vertebra showed that the OVX and OVX+PLF groups had a significantly lower BMD, BV/TV, Tb. Th, and Tb.N, along with higher Tb.Sp, compared to the Sham. Compared to the OVX+PLF group, significantly higher BMD, BV/TV, Tb.Th, and Tb.N, and lower Tb.Sp, were seen in the OVX+PLF+Dmab group (Fig. 3b–f).

**Fig. 4:**
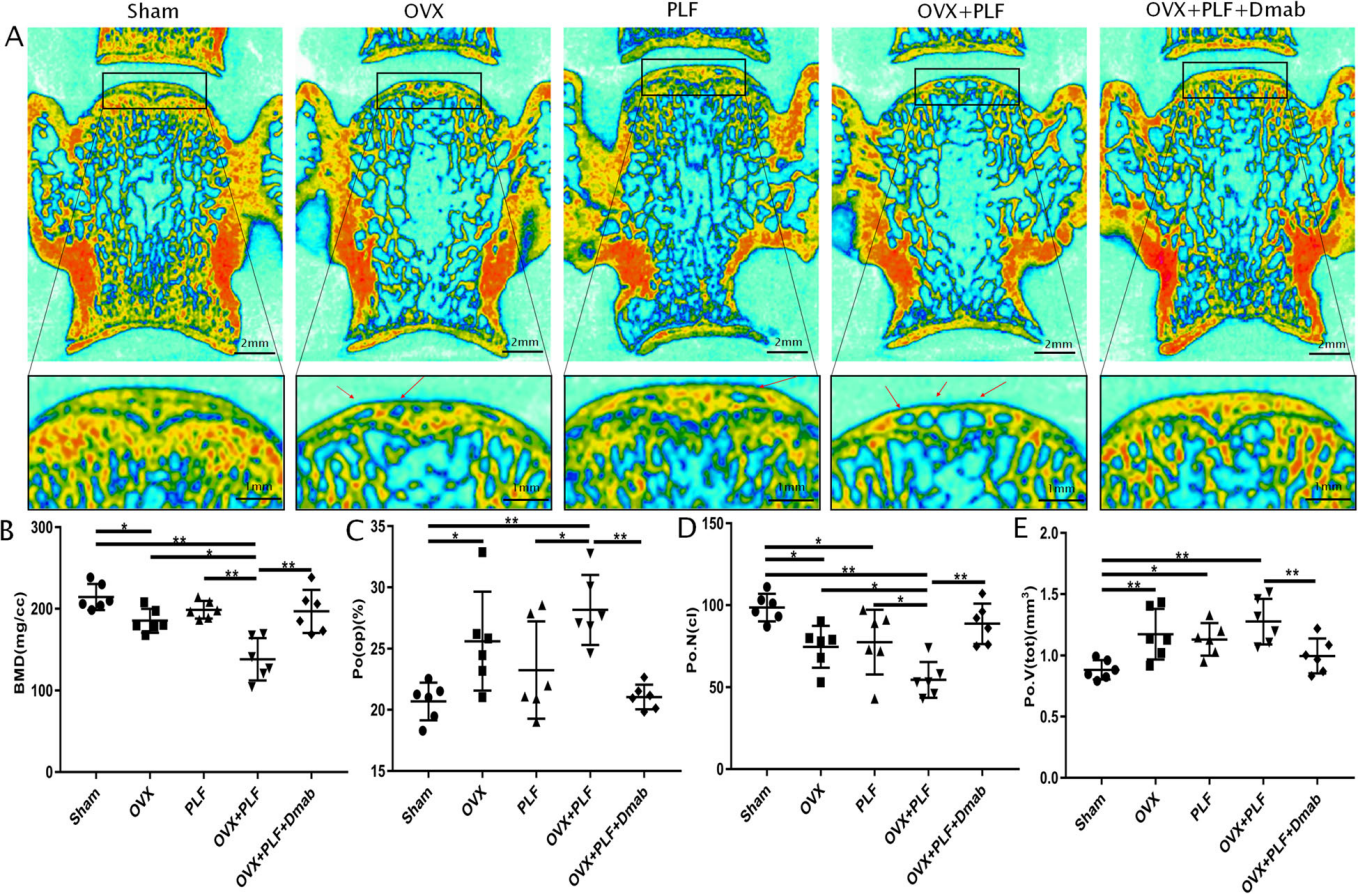
Changes in microarchitecture and porosity of L5/6 caudal endplate quantified by μCT analysis. Representative coronal images and parameters of caudal endplate in all groups (**a**). More cavities in OVX and OVX+PLF rats (red arrow) indicate osteochondral endplate remodeling. **b**–**e** Compared with the Sham group, a markedly lower number of closed pores (Po.N(cl)), higher open porosity (Po(op)), and total pore volume (Po.V(tot)) occurred in the OVX and OVX+PLF groups. The OVX+PLF+Dmab group showed a significantly higher number of closed pores, and lower open porosity (Po(op)) and total volume of pore space (Po.V(tot)) compared to the OVX+PLF group. Note: ^*^P < 0.05, ^**^P < 0.01; scale bars = 2 mm and 1 mm as indicated

In addition, a large number of cavities were found in the endplates of the OVX and OVX+PLF groups (Fig. 4a). To determine the degree of porosity in the endplate, μCT parameters of L5–L6 caudal endplates were examined. Compared to the Sham group, the OVX and OVX+PLF groups exhibited significantly lower BMD, and closed pores number, but significantly higher open porosity and total pore volume, all of which reflect higher endplate porosity. However, compared to the OVX+PLF group, the OVX+PLF+Dmab group had a significantly higher BMD, and number of closed pores, as well as lower open porosity and total pore volume (Fig. 4b–e). These results showed that Dmab treatment delayed the reduction of DHI and inhibited vertebral osteoporosis and osteochondral remodeling of the endplate.

### The evaluation of vertebral mechanical properties

**Fig. 5:**
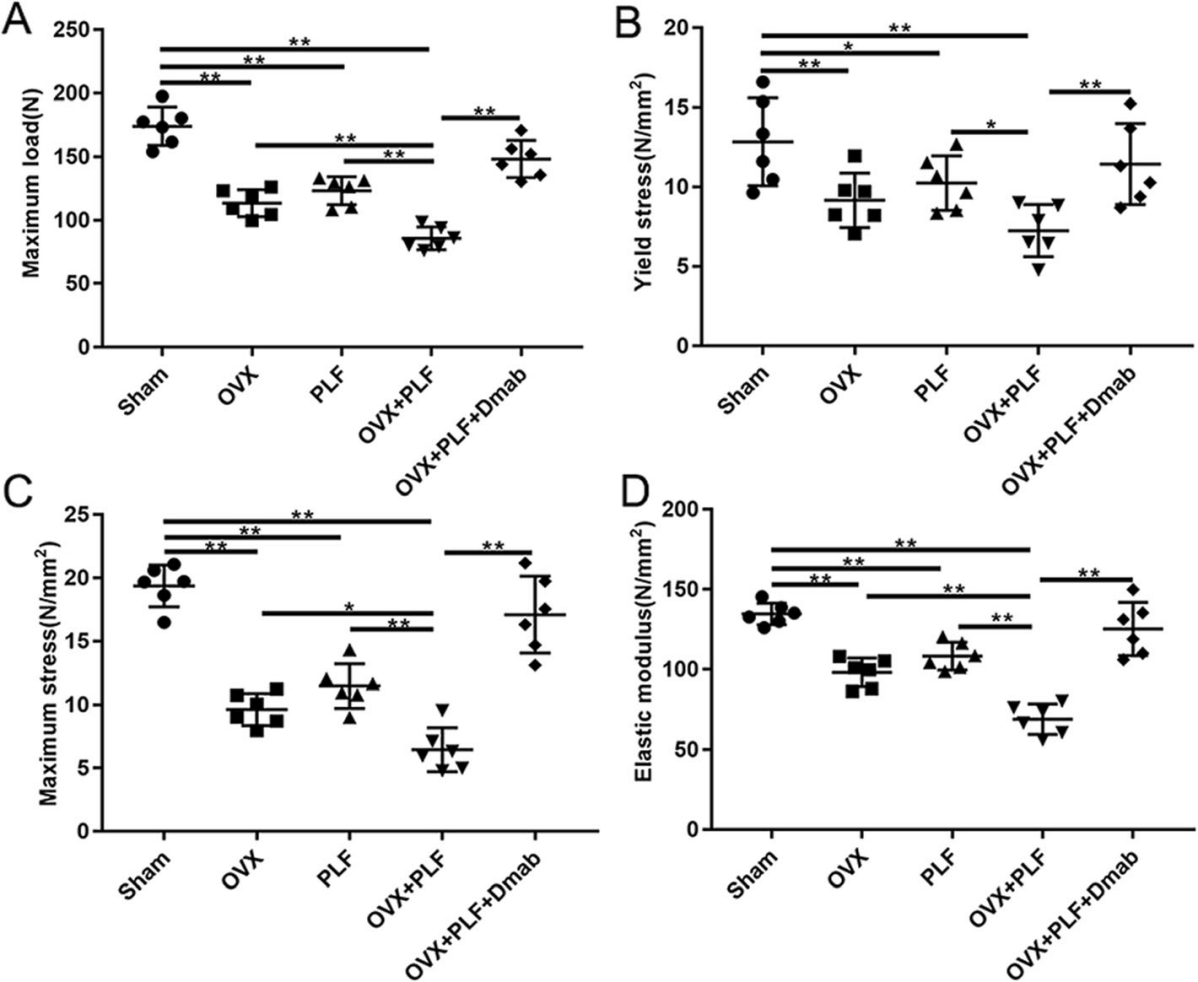
Results of L6 vertebral mechanical experiment in all groups (**a**–**d**). Compared to the Sham group, markedly lower maximum load, yield stress, maximum stress, and elastic modulus values were found in the OVX and OVX+PLF groups. Dmab treatment increased the above indices significantly compared to the OVX+PLF group. Note: ^*^P < 0.05, ^**^P < 0.01

To detect changes in the mechanical properties of adjacent vertebrae, we performed compression tests on L6 segments. Maximum load, yield stress, maximum stress, and elastic modulus values were significantly lower in the OVX, PLF, and OVX+PLF groups compared to the Sham group. However, Dmab treatment significantly improved the mechanical parameters, compared to the OVX+PLF group (Fig. 5a–d). These data were in accordance with the results of the μCT analysis, verifying the anti-osteoporosis effects of Dmab.

### Histological analysis of IVDs between L5 and L6 segments

**Fig. 6:**
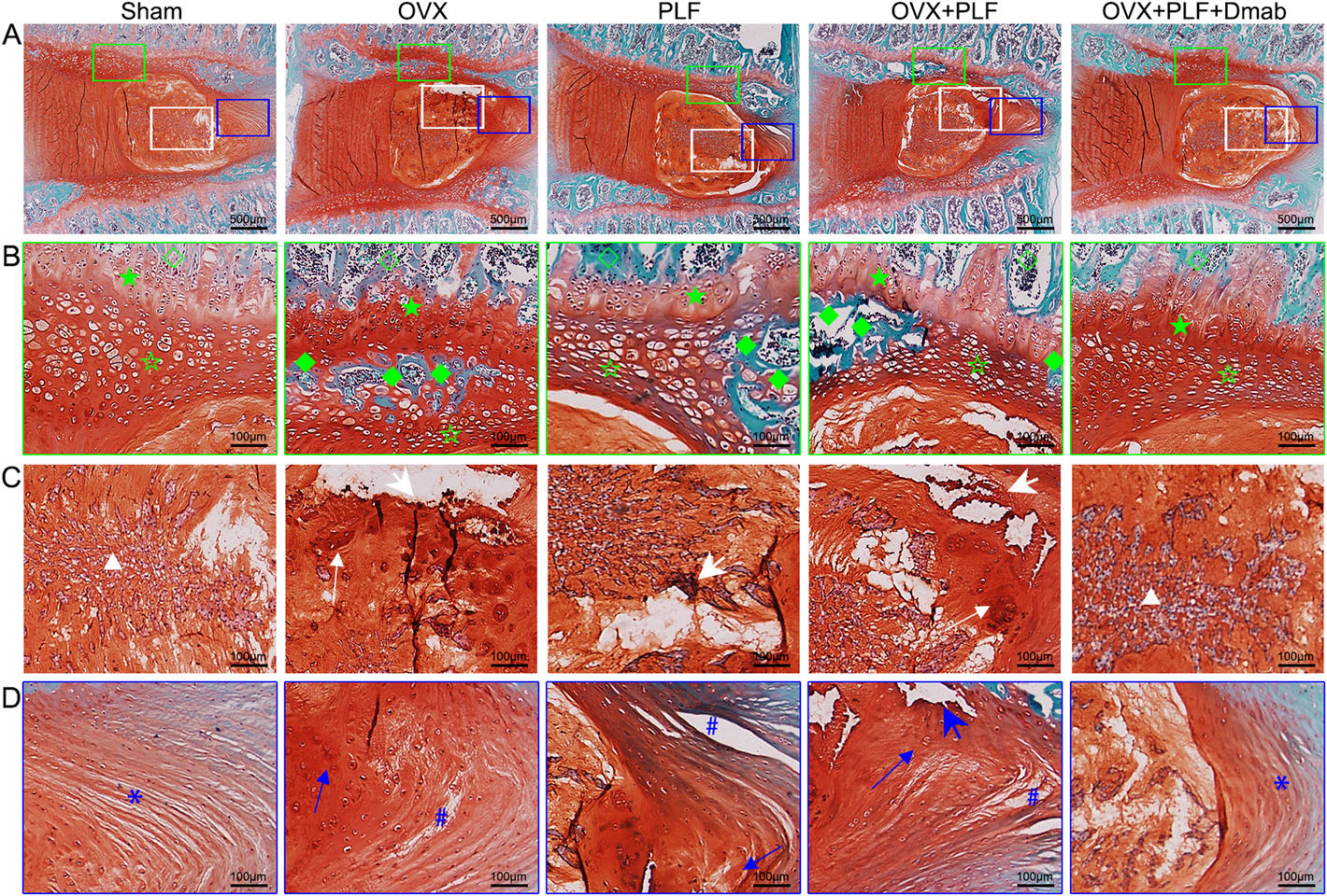
Histological illustration of the L5–L6 segments of the lumbar spine in different groups. **a** Safranin O and fast green staining of L5–L6. **b** Safranin-O green staining of cartilage endplate. Green hollow pentagram indicates cartilage endplate cells, and green solid quadrilateral indicates new bony tissue. **c** The degenerative changes in the nucleus pulposus visualized with safranin-O green staining. A white triangle indicates notochordal cells, thin white arrow indicates chondrocyte-like cells, and large white arrow indicates mucoid degeneration. **d** Safranin O and fast green staining of annulus fibrosus (blue asterisk indicates regular annulus fibrosus, blue pound sign indicates cleft/crack, thin blue arrow indicates chondrocyte-like cells, and large blue arrow indicates mucoid degeneration in the annulus fibrosus). Note:★, vertebral epiphysis; ☆, cartilage endplate; ◇, vertebral physis; ◆, bony tissue; scale bars = 500 μm and 100 μm as indicated

**Fig. 7:**
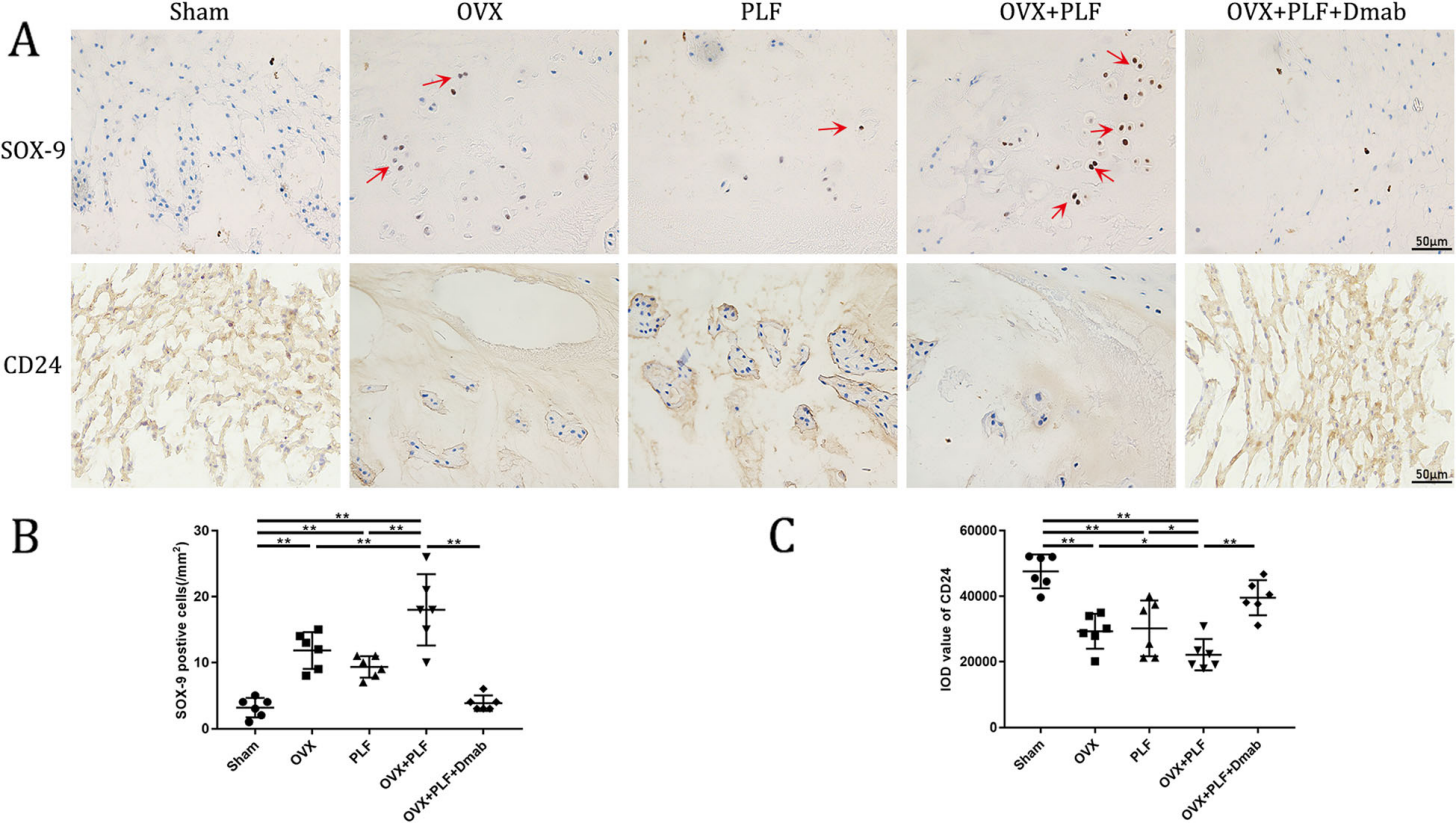
Immunohistochemistry assay for **a** CD24 and SOX-9 in the nucleus pulposus in different groups. The number of chondrocyte-like cells was found by counting the number of SOX-9-positive staining cells. The thin red arrow indicates chondrocyte-like cells. **b**, **c** Immunohistological analysis showed that the expression of CD24 in the OVX+PLF+Dmab group was greater than that in the OVX+PLF group. There were fewer SOX-9-positive cells in the OVX+PLF+Dmab group than in the OVX+PLF group. Note: *P < 0.05, **P < 0.01; scale bars = 50 μm as indicated

**Fig. 8:**
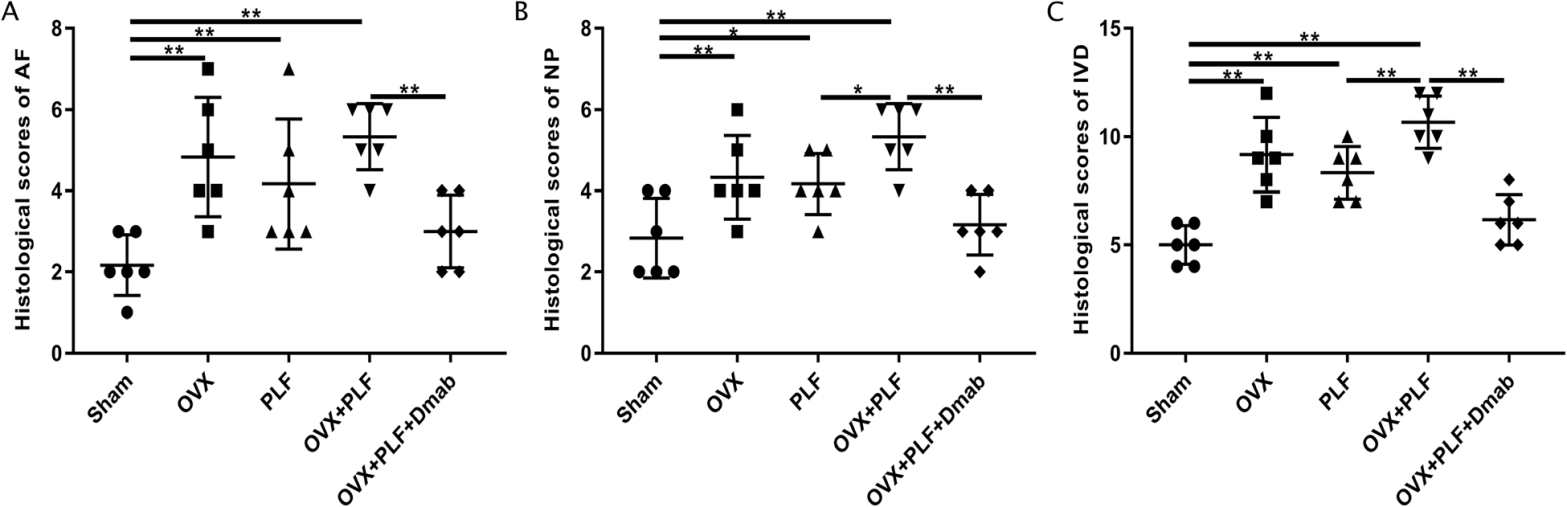
Histological scores of the L5–L6 intervertebral disc in different groups. **a** The histological scores of AF. **b** The histological scores of NP. **c** The histological scores of IVD. Note: ^*^P < 0.05, ^**^P < 0.01

To detect differences in the tissue structure of IVDs and changes in cell types in NP among the groups, we performed safranin-O green staining and immunohistochemical staining. Figure 6 shows that the structure of the intervertebral disc was intact in the sham group. Specifically, NP contained abundant notochordal cells accompanied by rich gel-like tissue (extracellular matrix), and AF annulus was arranged regularly and tightly. The boundary between the NP and AF was clear. Hyaline cartilage of the endplate contained chondrocytes. In contrast, intervertebral disc degeneration occurred in the OVX group, PLF group, and OVX+PLF group to varying degrees. CD24 were notochordal cell markers and SOX-9 was chondrocyte marker. The cell phenotypes of NP were classified based on CD24 and SOX-9 immunopositivity. As shown in Fig. 7, there was significantly less CD24 expression than in the sham group and there were significantly more SOX-9-positive cells in the OVX, PLF, and OVX+PLF groups than in the sham group, especially in the OVX+PLF group. However, Dmab treatment significantly upregulated the expression of CD24 and downregulated the number of SOX-9-positive cells relativere to the OVX+PLF group. These results showed that fewer notochord cells but more chondrocyte-like cells in the OVX, PLF, and OVX+PLF groups than in the sham group, and the structural composition of the NP was also different. Moreover, some notochordal cells were replaced by clustered doublets of chondrocyte-like cells, and the matrix around notochordal cells had varying degrees of mucoid degeneration and uneven distribution. At the same time, the fibrous annulus was broken and disordered. The calcification remodeling in the cartilage endplate increased, the AF annulus was broken and destroyed, and the intervertebral disc was occupied by disorganized fibrochondral tissue. These degenerative changes were the most obvious in the OVX+PLF group. Compared to the PLF group, IVD degeneration was more severe in the OVX+PLF group, which confirmed the negative effect of OVX in ASDD. Compared to the OVX+PLF group, pathological changes were effectively controlled in the OVX+PLF+Dmab group: the number of notochordal cells in the NP was increased, the number of chondroid cells was reduced, the degree of matrix myxoid degeneration was reduced, and AF arrangement of fibers was more orderly. AF, NP, and IVD scores confirmed these histological findings (Fig. 8).

### The turnover and remodeling of the vertebral body and endplate cartilage

**Fig. 9:**
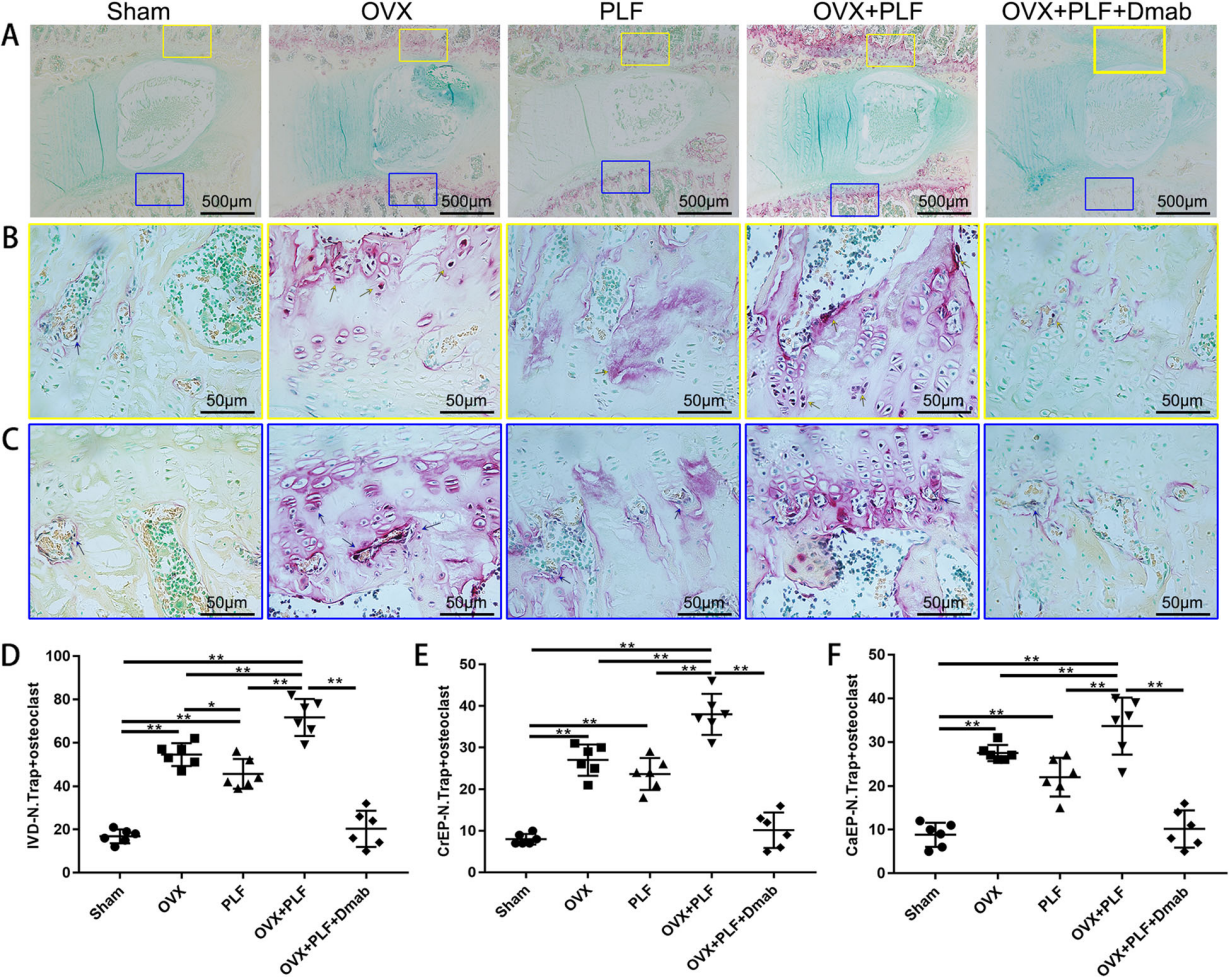
Tartrate acid phosphatase (TRAP) staining of L5/6. The osteoclasts were obtained by counting the number of TRAP-positive cells (N. Trap+). **a** A few TRAP-positive cells were distributed on the surface of the trabeculae of the subchondral bone and were rarely detected in the endplates of Sham and OVX+PLF+Dmab groups. However, TRAP-positive cells were significantly more frequent in the subchondral bone and the endplate of the OVX+PLF group, suggesting reduced osteoclast activity after Dmab treatment. **b** TRAP staining of CrEP (purple; yellow arrows). **c** TRAP staining of CaEP (purple; blue arrows). **d**–**f** Quantitative analysis of TRAP-positive cells for IVD, CrEP, and CaEP. Note: ^*^P < 0.05, ^**^P < 0.01. IVD intervertebral disc, CrEP cranial endplate, CaEP caudal endplate; scale bars = 500 μm and 50 μm as indicated

To evaluate osteochondral remodeling, TRAP staining was performed. As expected, the results were compatible with the results of μCT and safranin-O green staining, suggesting that excessive bone and cartilage remodeling occurred due to OVX or PLF. The OVX group had a larger number of osteoclasts in cranial and caudal cartilaginous EP compared to the Sham group, which illustrated the comprehensive effects of the OVX on bone and cartilage turnover. However, osteoclasts were observed only in the dorsal portion of cartilaginous EP near the lumbar fusion (the spinous process adjacent to L4–L5 on the dorsal side of the spine) in the PLF group, where mechanical stress might have aggregated due to uneven forces caused by lumbar fusion. The combined effects of OVX and PLF resulted in the distribution of a large number of osteoclasts on the trabecular surface of the subchondral bone and cartilaginous EP. After Dmab treatment, the number of osteoclasts was significantly lower (Fig. 9). These results showed that Dmab treatment inhibited turnover and remodeling of the vertebral body and endplate cartilage.

### Composition changes of the NP at the protein level

**Fig. 10:**
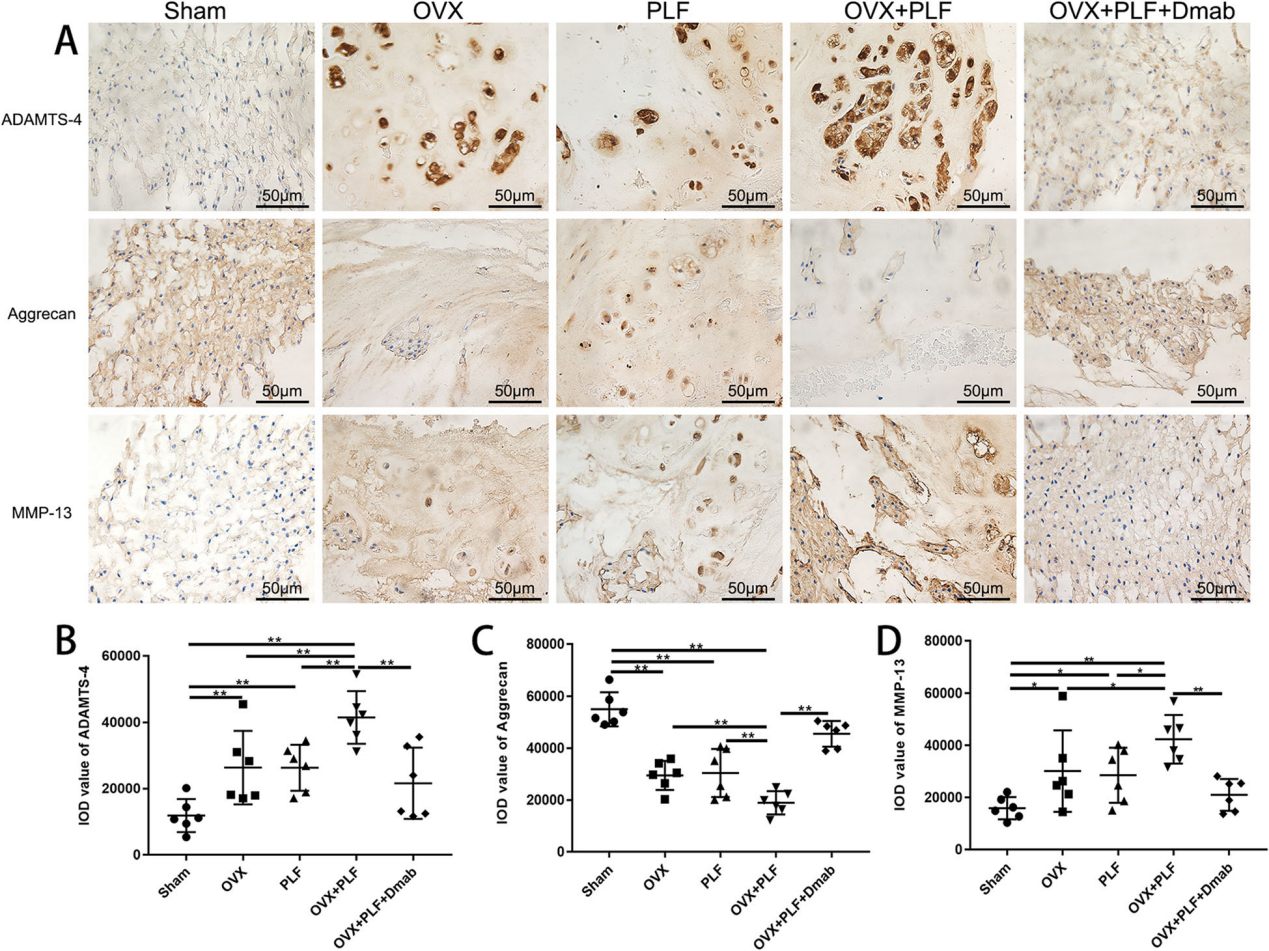
Immunohistochemistry assay for **a** ADAMTS-4, aggrecan, and MMP-13 in the nucleus pulposus in different groups. **b**–**d** Immunohistological analysis showed that the protein expression of aggrecan in the OVX+PLF+Dmab group was higher than that in the OVX+PLF group. MMP-13 and ADAMTS-4-positive staining in the nucleus pulpous were weaker in the OVX+PLF+Dmab group compared to the OVX+PLF group. Note: ^*^P < 0.05, ^**^P < 0.01; scale bars = 50 μm as indicated

Expression of aggrecan, MMP-13, and ADAMTS-4 was detected by immunohistochemistry to assess the matrix metabolism (Fig. 10a). In general, gelatinous NP is the main functional component of the IVD, and degeneration of the NP is regarded as a crucial aspect of IVD degeneration .In the Sham group, the NP structures showed strong immunoreactivity for aggrecan and weak immunoreactivity for MMP-13 and ADAMTS-4. In contrast, the NP showed weak aggrecan immunostaining and strong MMP-13 and ADAMTS-4 immunoreactivity in the OVX, PLF, and OVX+PLF groups, especially in the OVX+PLF group. However, the OVX+PLF+Dmab group showed stronger immunostaining for aggrecan and weaker immunostaining for ADAMTS-4 compared to the OVX+PLF group (Fig. 10b–d). These results suggested that Dmab may delay the progression of ASDD by changing the content of matrix components in the NP.

### Composition changes of NP at the mRNA level

**Fig. 11:**
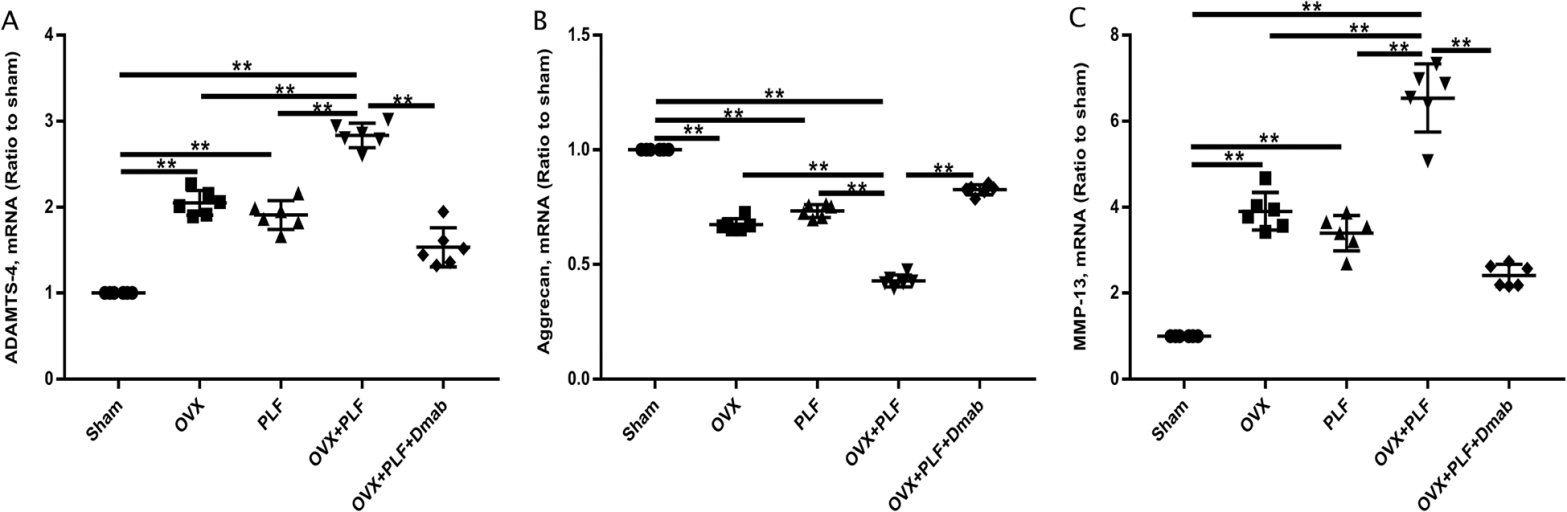
mRNA expression of ADAMTS-4, aggrecan, and MMP-13 in different groups. Note: Note: ^*^P < 0.05, ^**^P < 0.01

To quantify mRNA expression of aggrecan, MMP-13, and ADAMTS-4 in NPs, the NP tissues of each group were isolated and underwent RT-PCR testing. Compared to the Sham group, the OVX, PLF, and OVX+PLF groups showed lower aggrecan levels and higher MMP-13 and ADAMTS-4 mRNA levels. The OVX+PLF+Dmab group showed higher aggrecan levels and lower MMP-13 and ADAMTS-4 mRNA levels compared with the OVX+PLF group. These results were consistent with the finding from immunohistochemical staining (Fig. 11).

## Discussion

Considering the high morbidity of ASDD and the limitations of current treatment modalities, there is an urgent need to develop a treatment method that delays or even reverses ASDD and preserves the physiological function of the IVD [20, 21]. In this study, we demonstrated that Dmab treatment improved the prognosis of lumbar fusion by inhibiting vertebral osteoporosis and endplate cartilage remodeling, which also prevented ASDD. These effects of Dmab are beneficial for maintaining the structural and functional integrity of the adjacent vertebral body and endplates, which ensures the patency of the IVD’s nutritional pathway. In addition, Dmab preserved the normal structure and function of the intervertebral disc by regulating the metabolism of the extracellular matrix in the NP of the IVD.

To further explore the pathogenesis of ASDD, we passed steel wire through the bases of the spinous processes of L4 and L5 to fix them together. The fragments of the ilium were inserted between the transverse processes of L4 and L5 to simulate spinal fusion in postmenopausal women. X-ray examination showed that the fusion of the L4 and L5 segments was good in the PLF, OVX+PLF, and OVX+PLF+Dmab groups, and no activity was detected by manipulation. Microstructural analysis revealed significantly lower DHI in the OVX+PLF group than in the Sham group. The histological score of Safranin-O green staining in the OVX+PLF group was significantly higher than that in the Sham group. The immunohistochemical staining and RT-PCR results of NP tissue confirmed that, compared to the Sham group, the expression level of aggrecan was significantly lower in the OVX+PLF group, while the expression levels of ADAMT-4 and MMP-13 were significantly higher. These results are largely consistent with the results of previous studies [2, 3, 22].

In accordance with our previous study, we found a strong correlation between bone mass reduction and IVD degeneration in ovariectomized rats, which may be related to deterioration of the integrity and function of adjacent structures. Analysis of endplate cartilage remodeling confirmed that microstructure remodeling of subchondral bone caused by osteoporosis may further aggravate osteoarthritis and that the inhibition of subchondral bone remodeling could effectively delay the progression of osteoarthritis. It is suggested that subchondral bone remodeling is an indispensable factor in osteoarthritis development. The structures of joints and IVDs are similar [23–25]. To further investigate the relationship between vertebral osteoporosis and subchondral endplate bone remodeling and ASDD aggravation, we performed the μCT analysis of L5–L6 segments in the experimental ASDD model. The results showed obvious focal fragmentation and sparse intersecting bone trabecular areas, as well as varying degrees of endplate calcification. The vertebral Tb.Th, Tb.N, and BMD decreased significantly, while Tb.Sp increased significantly, suggesting that the subchondral bone structure changed; the endplate BMD and the number of closed pores significantly decreased whereas open porosity and total pore volume increased significantly, suggesting that the subchondral bone microstructure of the endplate was remodeled. It is worth noting that severe endplate calcification was observed in the OVX, PLF, and OVX+PLF groups, and surprisingly, there was also some degree of endplate calcification in the Sham group, suggesting that a certain degree of calcification of the endplate cartilage occurs under natural growth conditions.

In addition, vertebral osteoporosis after lumbar fusion is not only an important risk factor for postoperative vertebral nonunion complications but also leads to an increased bone conversion rate and biomechanical changes, which result in microenvironmental changes, thereby accelerating the development of ASDD [2, 26–28]. We performed TRAP staining on histological sections and found an increased number of osteoclasts, suggesting a high conversion rate of bone and cartilage at the intervertebral disc and vertebral interface in the ASDD model. At the same time, we conducted compression experiments on L6 vertebrae and found that, compared to the Sham group, the ultimate load (maximum load), yield stress, and elastic modulus of L6 vertebrae were significantly lower in the OVX and OVX+PLF groups, especially in the OVX+PLF group, suggesting a reduced trabecular hardness, increased trabecular brittleness, and altered vertebral biomechanics in the ASDD model.

In this study, we performed subcutaneous injection of Dmab. Dmab can effectively block the binding of NF-kB ligand receptor activator (RANKL) to its osteoclast-derived receptor (RANK), thereby inhibiting osteoclast-mediated bone resorption (osteoclast formation, activation, and survival) and promoting bone formation [1, 29]. During a 36-month follow-up, Cummings et al. [30] found that Dmab treatment in postmenopausal women could increase vertebral BMD and reduce the risk of vertebral fracture. In this study, we showed improved subchondral bone structure after Dmab treatment compared to the OVX+PLF group (higher BMD, BV/TV, Tb.Th, and Tb.N; lower Tb.Sp of the vertebral body). Ide et al. [1] confirmed that in addition to increasing BMD, Dmab also effectively improved lumbar fusion. Indeed, we found that the X-ray fusion score of the OVX+PLF+Dmab group was significantly higher than that of the OVX+PLF group, and observed greater new bone formation and higher X-ray optical density at the fusion site of L4-L5, confirming that Dmab could promote bone formation and facilitate the fusion of lumbar vertebrae. Dempster et al. [13] demonstrated that Dmab maximally preserved femoral neck cartilage remodeling while retaining modeling-based bone formation, and BMD increased continuously over time. The μCT analysis of the endplate showed that after treatment with Dmab, the endplate’s BMD and number of closed pores were significantly higher, while open porosity and total pore volume were significantly lower than those of the OVX+PLF group. The results of TRAP staining showed a large number of osteoclasts in the cartilage endplate, bone endplate, and the junction between the endplate and vertebral body in the OVX+PLF group, but the number of osteoclasts was significantly lower after Dmab treatment. This confirmed that Dmab could reduce bone conversion rate, improve the subchondral bone microstructure of the endplate, and inhibit remodeling of endplate cartilage. Based on the theory that IVD is dependent on endplate permeability, some studies have suggested that an increase in endplate porosity may delay IVD [31, 32]. However, more studies confirmed that the increase in porosity caused by endplate cartilage remodeling may be related to inflammation, excessive antigen exposure, and decreased intervertebral disc osmotic pressure, which further aggravate intervertebral disc degeneration [6, 33]. This argument has been confirmed again by the experimental results of this study. These changes may be related to the characteristics of Dmab (inhibition of osteoclast formation and activation, inhibition of osteoclast-mediated bone resorption, promotion of bone formation, and maintenance of the integrity of vertebral and endplate cartilage and subchondral microstructure and function).

IVDs function as a shock absorber system, and the AF and NP ensure flexibility to withstand mechanical loading, which can transfer loads and dissipate energy imposed on the spine [34]. During ASDD development, the biomechanical changes of the vertebrae hamper the dynamic balance of the spine [35]. Sakai and Grad [36] suggested that abnormal IVD biomechanical changes can lead to local tissue injury, and altered distribution of the disc’s extracellular matrix and microenvironment, eventually leading to IVD degeneration. Our study revealed that, compared to the OVX+PLF group, the maximum load, yield stress, maximum stress, and elastic modulus of the L6 vertebral body increased after Dmab treatment, highlighting the ability of Dmab to effectively maintain the biomechanical properties and improve the trabecular structure of the vertebral body. Changes and disorders in the IVD matrix are the basic characteristics of IVD degeneration [37, 38].

**Fig. 12:**
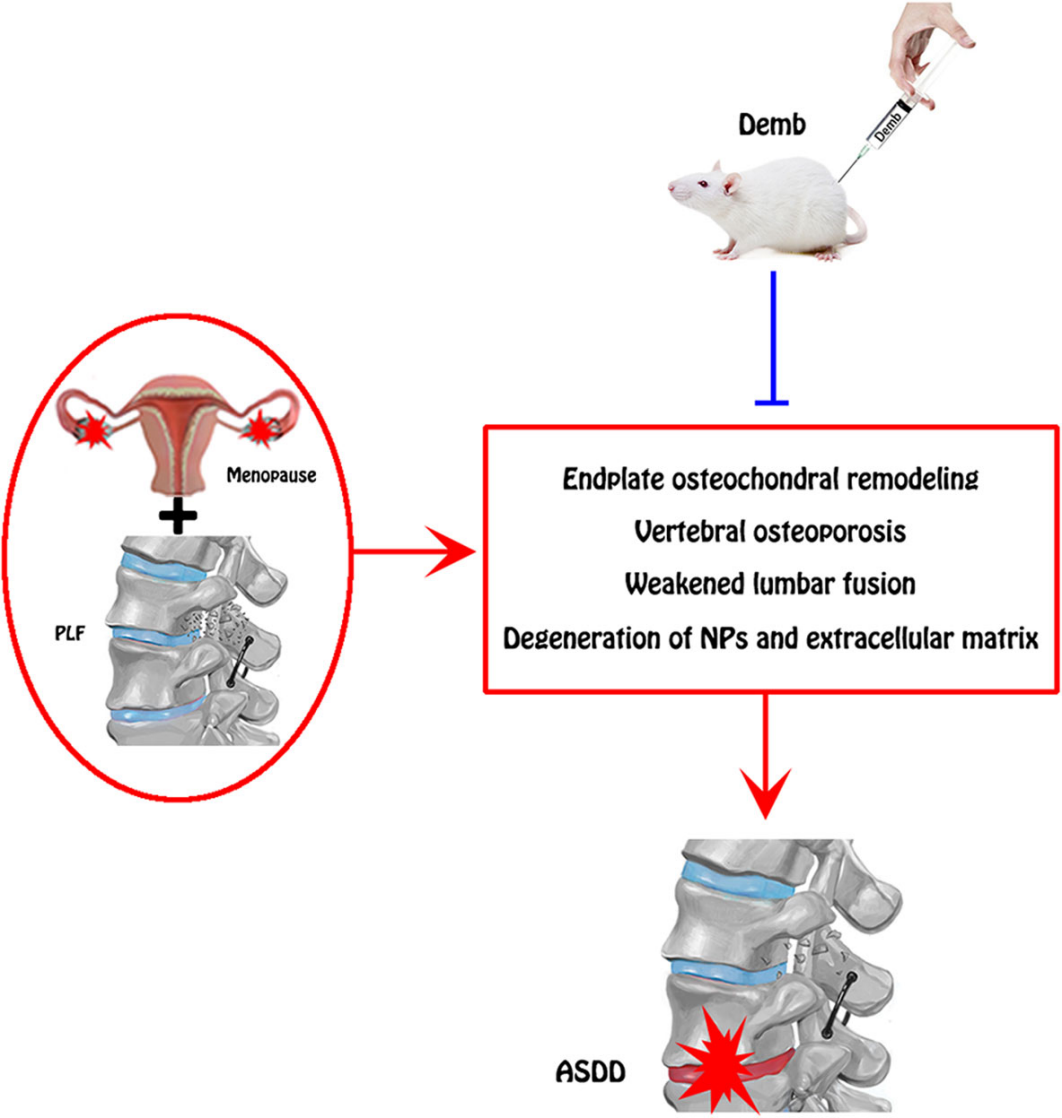
Potential mechanism of Dmab in ASDD treatment in rats. Dmab enhances lumbar fusion and alleviates ASDD in NP cells via suppressing endplate osteochondral remodeling, vertebral osteoporosis, and extracellular matrix degradation

In general, NP cells produce extracellular matrix including collagen II and proteoglycans, which are the main components of the gelatinous tissues of NP [39]. The gelatinous NP is essential for disc function, and degeneration of the NP is regarded as a crucial component of IVD degeneration [40]. In order to evaluate the metabolic status of the IVD matrix, we detected the expression of metabolic markers via immunohistochemical staining and PCR. The results showed the expression of aggrecan was significantly increased while the expressions of ADAMT-4 and MMP-13 were significantly decreased after treatment with Dmab compared to the OVX+PLF group. Safranin-O green staining showed that the structural composition of NP changed significantly in the OVX+PLF group; some NP cells were replaced by chondrocyte-like cells distributed in clusters, and the matrix around NP cells had different degrees of myxoid degeneration, uneven distribution, fibrous annulus fracture, and disordered arrangement, all of which were improved in the OVX+PLF+Dmab group (Fig. 12). In this context, one limitation of the present study is lack of well-accepted markers with high specificity for notochord cells. Here, we analyzed the expressions of CD24 and Sox-9, as markers for the notochord cells [41] and chondrocyte-like cells [42] and their relationships to distinct cell morphologies in NP. We also confirmed the results of Safranin-O green staining. Further studies are needed to confirm the results for cell type identification.

## Conclusions

In summary, we demonstrated for the first time that ASDD induced by OVX and PLF in a rat model could be retarded by subcutaneous Dmab injections. The protective effects of Dmab primarily acted by maintaining the integrity of structure and function by inhibiting vertebral osteoporosis and endplate cartilage remodeling. These findings could provide a basis for a novel therapeutic strategy for ASDD. However, our observations are still preliminary. Future studies should focus on the Dmab dose and cycle, and controlled trials should be designed to evaluate the effects of Dmab in a dose-dependent and time-dependent manner.

## Data Availability

The datasets used and/or analyzed during the current study are available from the corresponding author on reasonable request.

## Abbreviations

BMD: Bone mineral density
ASDD: Adjacent segmental intervertebral disc degeneration
Dmab: Denosumab
μCT: Microcomputed tomography
IVD: Intervertebral disc
DHI: Disc height index
OVX: Ovariectomy
PLF: Posterolateral lumbar fusion
Agg: Aggrecan
MMP-13: Metalloproteinase-13
ADAMTS-4: A disintegrin and metalloproteinase with thrombospondin motifs 4
PO(op): Open porosity
Po.N(cl): Number of closed pores
Po.V(tot): Total volume of pore space
ROI: Region of interest
BV/TV: Bone volume fraction
Tb.N: Trabecular number
Tb.Sp: Trabecular separation
Tb.Th: Trabecular thickness
TRAP: Tartrate-resistant acid phosphatase
RT-PCR: Real-time polymerase chain reaction
AF: Annulus fibrosus
EP: Endplates
