# Correction to: Denosumab alleviates intervertebral disc degeneration adjacent to lumbar fusion by inhibiting endplate osteochondral remodeling and vertebral osteoporosis in ovariectomized rats

**DOI:** 10.1186/s13075-021-02601-z

**Published:** 2021-09-09

**Authors:** Qi Sun, Fa-Ming Tian, Fang Liu, Jia-Kang Fang, Yun-Peng Hu, Qiang-Qiang Lian, Zhuang Zhou, Liu Zhang

**Affiliations:** 1 Department of Orthopedic Surgery, Hebei Medical University, Shijiazhuang, People’s Republic of China; 2 Medical Research Center, North China University of Science and Technology, Tangshan, People’s Republic of China; 3 Department of Bone and Soft Tissue Oncology, The Third Hospital of Hebei Medical University, Shijiazhuang, People’s Republic of China; 4 Department of Orthopedic Surgery, Hebei Medical University, 361 Zhongshan ERoad, 050000 Shijiazhuang, Hebei People’s Republic of China

**Correction to: Arthritis Res Ther 23, 152 (2021)**

**https://doi.org/10.1186/s13075-021-02525-8**

Following publication of the original article [1], the authors reported an error in Fig. 1b wherein a wrong version was published. The correct figure is presented below.

**Fig. 1:**
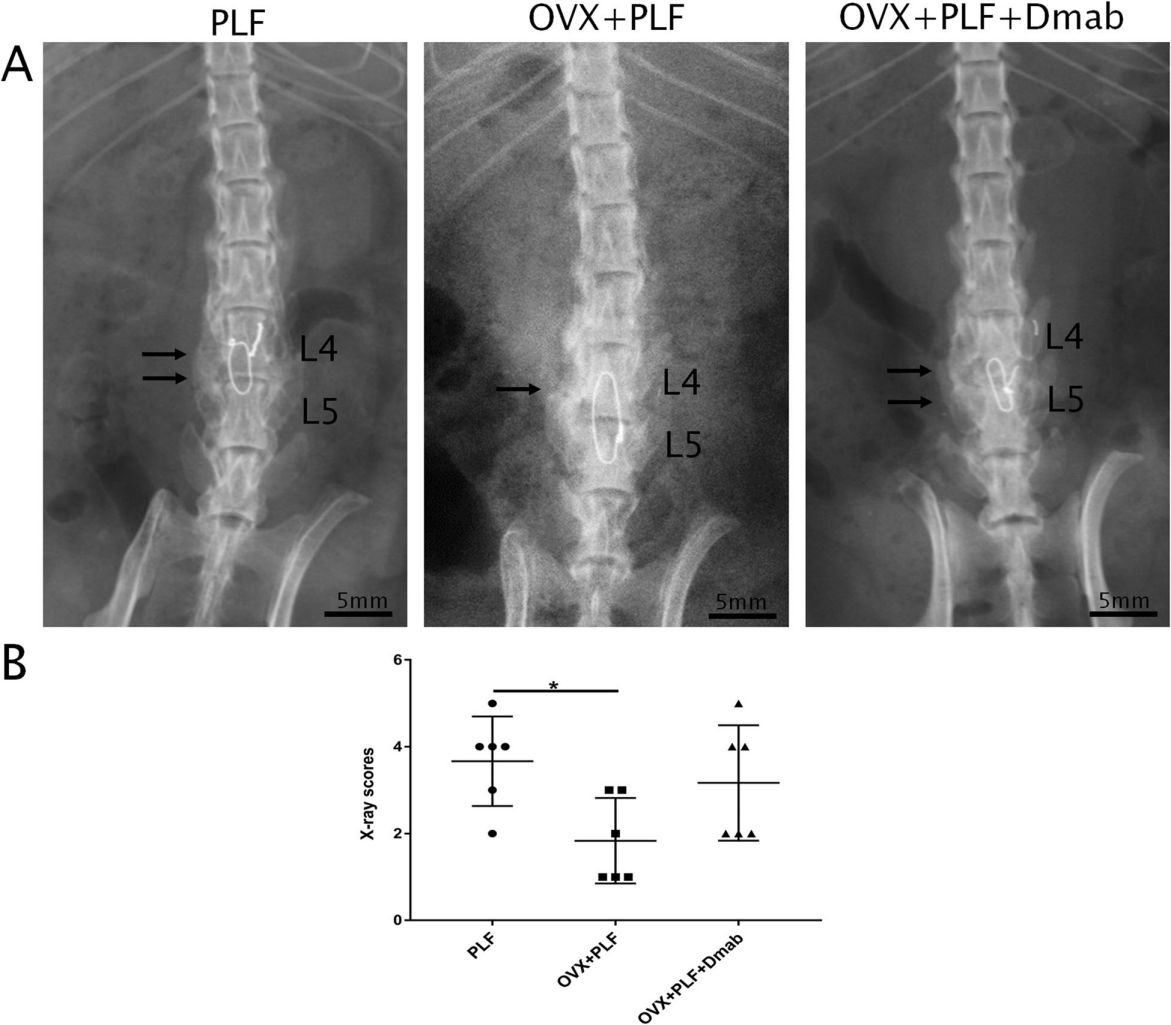
Radiographic evaluation of lumbar fusion at 4 weeks post-PLF. **a** Representative radiographic images from the three groups. PLF of the L4–L5 segments was performed via intertransverse process fusion with an autologous iliac bone graft and spinous-process wire fixation. Compared with the OVX + PLF group, the OVX + PLF + Dmab group showed higher radiographic density with more new bone formation at the fusion site (thin arrow indicates new bone formation). **b** X-ray scores of lumbar fusion. Note: *P < 0.05; scale bars = 5 mm as indicated

The original article [1] has been updated.
